# Correlation of serum C-reactive protein, white blood count and neutrophil percentage with histopathology findings in acute appendicitis

**DOI:** 10.1186/1749-7922-7-27

**Published:** 2012-08-06

**Authors:** Shefki Xharra, Lumturije Gashi-Luci, Kumrije Xharra, Fahredin Veselaj, Besnik Bicaj, Fatos Sada, Avdyl Krasniqi

**Affiliations:** 1 Department of Surgery, Regional Hospital, Prizren, Kosovo; 2 Institute of Pathology, Faculty of Medicine, University of Prishtina, Prishtina, Kosovo; 3 Department of Microbiology, National Institute of Public Health of Kosovo, Prishtina, Kosovo; 4 Department of Urology, University Clinical Center of Kosovo, Prishtina, Kosovo; 5 Department of Abdominal Surgery, University Clinical Center of Kosovo, Prishtina, Kosovo; 6 Department of Anesthesiology, University Clinical Center of Kosovo, Prishtina, Kosovo

**Keywords:** Acute appendicitis, CRP correlation, White blood count, Neutrophil percentage, Histopathology findings

## Abstract

**Background:**

Acute appendicitis is one of the most common surgical emergencies. Accurate diagnosis of acute appendicitis is based on careful history, physical examination, laboratory and imaging investigation. The aim of the study is to analyze the role of C-reactive protein (CRP), white blood count (WBC) and Neutrophil percentage (NP) in improving the accuracy of diagnosis of acute appendicitis and to compare it with the intraoperative assessment and histopathology findings.

**Materials and methods:**

This investigation was a prospective double blinded clinical study. The study was done on 173 patients surgically treated for acute appendicitis. The WBC, NP, and measurement of CRP were randomly collected pre-operatively from all involved patients. Macroscopic assessment was made from the operation. Appendectomy and a histopathology examination were performed on all patients. Gross description was compared with histopathology results and then correlated with CRP, WBC, and NP.

**Results:**

The observational accuracy was 87,3%, as compared to histopathological accuracy which was 85.5% with a total of 173 patients that were operated on. The histopathology showed 25 (14.5%) patients had normal appendices, and 148 (85.5%) patients had acutely inflamed, gangrenous, or perforated appendicitis. 52% were male and 48% were female, with the age ranging from 5 to 59 with a median of 19.7. The gangrenous type was the most frequent (52.6%). The WBC was altered in 77.5% of the cases, NP in 72.3%, and C-reactive protein in 76.9% cases. In those with positive appendicitis, the CRP and WBC values were elevated in 126 patients (72.8%), whereas NP was higher than 75% in 117 patients (67.6%). Out of 106 patients with triple positive tests, 101 (95.2%) had appendicitis. The sensitivity, specificity, and positive predictive values of the 3 tests in combination were 95.3%, 72.2%, and 95.3%, respectively.

**Conclusion:**

The raised value of the CRP was directly related to the severity of inflammation (p-value <0.05). CRP monitoring enhances the diagnostic accuracy of acute appendicitis. The diagnostic accuracy of CRP is not significantly greater than WBC and NP. A combination of these three tests significantly increases the accuracy. We found that elevated serum CRP levels support the surgeon's clinical diagnosis.

## Introduction

Acute appendicitis is one of the most common surgical emergencies and the most common source of infection in community-acquired intra-abdominal infections [1-3]. Its diagnosis is usually made depending on the presenting history, clinical evaluation, and physical examination [1,2,4]. It is further reinforced by laboratory investigations, such as white blood cells, differential counts (the percentage of neutrophil granulocytes and band neutrophil granulocytes), and C-reactive protein (CRP) that were the only diagnostic tools for many years [4-10]. It has been estimated that the accuracy of the clinical diagnosis of acute appendicitis is only between 76 percent and 92 percent [9,11]. Thus, accurate diagnosis of acute appendicitis is still difficult [1,12,13]. The perforation rate is high, as well as the number of negative appendectomies [9,14]. Following the introduction of ultrasound scans during the last two decades and computed tomography (CT) in the last decade, the rate of negative appendectomies has decreased [4,15-17], but the perforation rate has remained high (22%-62%) [4,18,19]. Negative appendectomies are one of the burdens facing not only the general surgeon but also the patient her/himself and society as a whole, since appendectomy, as any other operation, results in socio-economic impacts in the form of lost working days and declined productivity.

CRP is a non-specific inflammatory marker that is used routinely in many hospitals as an aid in the diagnosis of patients with an acute abdomen [9,10,14]. An acute phase protein is produced in the liver. Normal serum concentration is less than 10 mg/l 8–12 hours after infection or trauma; the increase of acute phase protein in liver the CRP is more important in clinical practice. Production of CRP is controlled by Interleukin-6 and in a few minutes increases from 10 to 1,000 times. CRP is increased in infections, inflammatory arthritis, autoimmune disorders, neoplasia, pregnancy, and aging [9,10,20-24].

Many reports have investigated the value of the raised serum CRP measurement in improving the diagnosis of acute appendicitis [9,10,25].

Additional tests that would improve the diagnostic accuracy and reduce the number of unnecessary operations are needed. This is particularly important these days when health planning is driven by cost containment. The C-reactive protein (CRP), together with other acute-phase proteins, increased in response to tissue injury [26].

The aim of this study was to analyze the role of C-reactive protein (CRP) values, in accuracy of diagnosis of acute appendicitis in comparison with WBC, NP, the surgeon's clinical diagnosis, and the histopathologic findings.

## Patients and methods

### Patients

The study included randomly all operated patients (173) suspected of acute appendicitis between November 2008 and February 2009 in the Department of Surgery.

### Methods

Clinical signs of acute appendicitis determined by the surgeon and the duration of the symptoms were documented on admission. The clinical signs included direct tenderness in the right lower quadrant, percussion and rebound tenderness, localized rigidity, and diffuse rigidity of the abdominal wall. At least one clinical sign had to be present in order to consider the patient positive for clinical signs. In all operated patients, in-hospital observation time until the surgical procedure was performed was recorded. The surgeons were aware of the routine laboratory and ultrasound findings.

Blood samples for routine laboratory tests (white blood cell count, differential count), and C-reactive protein were obtained on admission. White blood cell and differential counts were measured by the Hematology Analyzer (HARIBA ABX Micros 60). The normal WBC value in our laboratory is 0–10 x 109/L. Levels above 10 x 109/L were considered as above normal. The percentage of neutrophils was considered elevated when >75%. The C-reactive protein concentration was quantified by a Latex agglutination slide test for the qualitative and semi-quantitative determination in Non-diluted serum (Humatex, Wiesbaden, Germany). For semi-quantitative determination, serum dilutions were prepared with the 0.9% sodium chloride, according to the instructions of the manufacturers. Each dilution was tested according to the qualitative procedure described above until no further agglutination was observed. The serum CRP concentration was then estimated by multiplying the dilution factor from the last dilution with visible agglutination (2, 4, 8, 16, 32) by the detection limit (6 mg/l). E.g. if the agglutination titer appears at 1:16, the approximate serum CRP level is 16 x 6 = 96 mg/l. The normal CRP level in our laboratory is 0–6 mg/L. Levels above 6 mg/L were considered as being above normal.

Serum CRP measurements were not taken into account for the decision of surgical intervention and to compare it with the surgeon's clinical diagnosis. Further, the laboratory staff was not informed about the clinical findings, decisions, and outcomes (double blind study).

Removed appendixes were fixed in 4% formalin, stained with hematoxylin and eosin (H&E) and analyzed histologically. Based on the histological features of the removed appendix, according to the criteria described by Shashtari M H S, 2006 (24), the patients were divided into three groups: Group A normal appendix, Group B inflamed appendix (simple appendicitis), and Group C perforated/gangrenous appendix (complicated appendicitis). The final diagnosis was based on the histology and, in the case of perforation, on the macroscopic evaluation by the surgeon. The pathologists were not informed of the patients’ clinical and laboratory data, except for the surgical diagnosis.

### Statistical analysis

All variables showing a significant difference between the groups were further analyzed. The receiver-operating characteristic (ROC) curves were drawn to define the optimum sensitivity, specificity, cut-off value, predictive values, and diagnostic accuracy, determined by the area under the ROC curve (AUC) of the studied laboratory markers.

## Results

Out of a total of 173 patients, the histopathologic findings confirmed acute appendicitis in 148 (85.55%) patients. Normal appendixes were removed in the remaining 25 (14.45%) patients: males were 52.02% (N = 90), females 47.97% (N = 83), and children 39.3% (N = 68). The male female ratio was 1.09:1. The age range was from 5 to 59 years with the mean (SD) being 19.7 years (± 10,5), whereas 83.5% of patients were under 30 years old. According to the histopathology reports, Group A where normal appendix was found comprised 25 (14.45%) patients, whereas inflamed appendix was found in 148 (85.5%) patients. Among patients with a positive appendicitis, 36 (20.81%) belonged to group Group B with acute simple appendicitis and 112 (64.74%) had a ruptured/perforated/gangrenous appendix (Group-C, complicated appendicitis). The rate of perforated appendicitis was 12.1% (Table 1).

**Table 1:** Distribution of histopathologic features of appendix by sex

**Histopathology of Appendix**		**Female**	**Male**	**N**	**%**
**Group - A** Normal appendix		20	5	25	14.5
**Group - B**	Catarrhal App.	2	0	2	1.2
(Non-complicated appendicitis)	Phlegmonous App.	23	11	34	19.7
**Group - C**	Gangrenous App.	31	60	91	52,6
(complicated appendicitis) Perforative App.		7	14	21	12,1
**Total**	**N**	83	90	173	100
	**%**	48	52	100	

Among the patients in Group A, the most common diagnoses associated with primary negative appendectomy included nonspecific abdominal pain 15 (8.7%), ruptured ovarian cysts 4 (2.3%), mesenteric lymphadenitis 5 (2.9%), and urinary infection 1 (0.6%).

In Group A the CRP values ranged from 0 to 96 with a mean of 10.6 mg/l. In Group B these values were from 0 to 192 with a mean value of 37 mg/l, and in Group C from 0 to 192 with a mean of 79.2 mg/l. The serum CRP levels were normal in 22 patients with acute appendicitis. Thus, the false-negative rate of CRP was 12.71 percent. Of the 25 patients with normal appendectomy, serum CRP levels were slightly elevated in 7 patients. A false-positive rate of CRP was 4.05 percent. Further, based on the surgeons’ clinical impression, the diagnosis was true in 87.28% (N = 151) and false in 12.72% (N = 22) patients. In the present study, the positive predictive value of the CRP was 94.7%, specificity 72%, sensitivity 85.1%, and accuracy 83.2%.

Similarly, when the WBC count was assessed, Group A varied from 5.3 to 14.7 (mean 8.8 x10^9^/l), Group B from 5.0 to 28.0 (mean 12.6 x10^9^/l), and Group C from 5.0 to 28.0 (mean 15.6 x10^9^/l). The false positives were 4.62% and false negatives were 12.72% with a sensitivity of 85.1% and a specificity of 68%,; the positive predictive value was 94% and the accuracy was calculated to be 82.6%. The neutrophil percentage in Group A varied from 54.2 to 88.6 (mean 71.5), in Group B from 56.2 to 94.3 (mean 79.8) and in Group C from 60.7 to 96.6 (mean 84.0). The false positives were 4.62% and false negatives 17.92% with a sensitivity of 79.1% and the specificity 68%; the positive predictive value was 93.6% and the accuracy was calculated to be 77.5%.

The WBC and CRP were elevated in 126 (85.1%) cases with positive histopathology (Groups B and C). Seven patients had normal CRP and eight patients had normal WBC. In 25 patients with negative appendix, 18 had a normal CRP and 17 had normal WBC; only 5 patients had both CRP and WBC values increase. Again, in patients in Groups B and C, 113 (76.35%) had both WBC and CRP value increase and 9 patients had both values in the normal range. Combining all three parameters (WBC, CRP and percentage of neutrophil count) had positive results for the appendicitis in 101 (68.24%) patients (Groups B and C), and only 5 patients had one or more values in the normal range. In Group A, only five patients had all the three values increase and 13 patients had one or more values in the normal range. The combined WBC and CRP had a sensitivity, specificity, and positive predictive value of 95.3%, 91.1%, and 95.8%, respectively. While the combined percentage of the neutrophil count and CRP had a sensitivity, the specificity and positive predictive value of 94.3%, 91.1%, and 95.2%, respectively. Combined all the three parameters (WBC, CRP, and percentage of neutrophil count) gave the sensitivity, and specificity of 95.3% and 91.9%, respectively. The positive predictive value was 95.3% (Table 2).

**Table 2:** Diagnostic accuracy, sensitivity, specificity, and positive (PPV) of white blood cell (WBC) count, C-reactive protein (CRP), percentage of neutrophil (PN) and combined WBC, CRP and PN in diagnosing acute appendicitis

**Indices of diagnostic values**				
**Diagnostic method**	**Diagnostic accuracy**	**Sensitivity (%)**	**Specificity (%)**	**PPV (%)**
**CRP**	83.2	85.1	72	94.7
**WBC**	82.6	85.1	68	94
**PN**	77.5	79.1	68	93.6
**CRP + LEU**	90.1	92.6	75	95.8
**CRP + PN**	91.1	94.3	72	95.2
**LEU + PN**	87.1	89.9	71.4	94.7
**CRP + LEU + PN**	91.9	95.3	91.9	95.3

## Discussion

The positive CRP is more accurate than the WBC and neutrophil counts and combined together it further improves diagnostic accuracy [10]. In a double blind study Asfar et al. (2000) reported a sensitivity and specificity of CRP as 86.6% and 93.6%, respectively. They concluded that a normal CRP value probably indicates a normal non-inflamed appendix [14]. It is a more sensitive test than the WBC and neutrophil counts and their combined usage significantly increases sensitivity and specificity. Erkassap (2000) in a positive study on 102 patients reported that sensitivity and specificity of the CRP were 96% and 78%, respectively; the positive predictive value was 100% [27]. In a retrospective study, Wu and coworkers (2005) concluded that the combined usage of the WBC, neutrophil count, and the CRP monitoring increased the positive predictive value [28]. Grönroos (1999) in his study concluded that when both the WBC and CRP are normal, acute appendicitis is very unlikely [29].

In our study, the rate of complicated appendicitis at admission to the hospital was very high (Table 1). 112 (64.7%) patients had a ruptured/perforated/gangrenous appendix. The rate of perforated appendicitis was 12.1%. The delayed treatment can be attributed to the fact that most of the rural patients either did not report to the medical practitioners during the early stages of the disease or were seen by sought expert medical care only when complicated. Misdiagnosis by qualified medical practitioners in rural places delayed the reporting of patients to surgery, treating them with as gastroenteritis, urinary infection, etc. In these regions, the primary healthcare systems are not well-established; missed and delayed diagnosis is a major factor in complicating appendicitis.

According to Shakhatreh (2000), CRP measurement is very useful in the diagnosis of acute appendicitis, but it does not replace the clinical judgment of a surgeon [11]. Accuracy of the CRP (83.2%) is not significantly greater than the WBC (82.6%) and NP (80%). A combination of these significantly increases the accuracy to 91.9%. Anderson (2000) in a prospective study on 420 patients with borderline diagnosis of appendicitis concluded that the WBC and neutrophil count are the better criteria for the subsequent examinations [23]. In our study, from 148 patients with acute appendicitis, 22 patients had CRP and WBC in the normal range (12.72%). Mean values of the CRP in simple acute appendicitis (Group-B) were significantly greater than in normal appendix (Group A) (p <0.001), and also in complicated acute appendicitis (Group C) the CRP is significantly greater than in normal appendix and uncomplicated acute appendicitis (p <0.0001). The WBC and neutrophil percentage are also increased in correlation with severity of inflammation (p >0.05). None of these tests are 100% diagnostic. The CRP measurement or leukocyte count by itself is not completely preventive for negative appendectomy [30]. A study on 200 children showed that unlike the adult, normal leukocyte and CRP does not rule out acute appendicitis in pediatric cases [31]. Our results showed that the most affected age group was 10–19 years old (50.3%). A significant difference regarding CRP values as being diagnostic tools of acute appendicitis for different age groups and genders was not found.

In our study, the CRP values corresponds to the series with high percentage of complicated appendicitis, which is typical for rural hospitals and dysfunctional healthcare systems. But, the consistence of CRP level with the severity of appendicitis was reported by the other authors as well [32].

There are in use different clinical classification for the acute appendicitis [32,33], but, since the correlation of CRP values with histopathology findings were studied, we used the classification that combines the gross appearance of the appendix with pathologic stage [33]. Actually, the non-surgical initial management of acute appendicitis with catarrhalis changes (inflammation within the mucous membrane), or phlegmonous changes (inflammation in all layers) has been shown to be safe and effective [34,35].

Our results and other studies as well [32,36], clearly suggested that CRP leads to precise prediction of the severity of acute appendicitis. We think that CRP is not specific test for appendicitis, therefore, before diagnostic decision and indication for treatment, clinicians must depend on structural interpretation of their subjective experience, clinical information and modalities such as laboratory tests, ultrasonography and computed tomography where is available [37,38]. Finally, laboratory tests combined with imaging diagnostic procedures, remains the useful tools in establishing the diagnosis of acute appendicitis and excluding other causes of acute abdominal pain.

## Conclusions

The diagnostic accuracy of the CRP is not significantly greater than the WBC and NP. The increased value of the CRP was directly related to the severity of the inflammation (p <0.05). The combination of the CRP, the WBC, and the neutrophil percentage has greater diagnostic accuracy in acute appendicitis. This preoperative combination significantly decreases false positive and false negative diagnosis, but none of these is 100% diagnostic for acute appendicitis.

We found that elevated serum CRP levels support the surgeon's clinical diagnosis. We recommend CRP measurement as a routine laboratory test in patients with suspected diagnosis of acute appendicitis.

## Competing interests

The authors declare that they have no competing interests.

## Authors’ contributions

SX designed the study, carried out acquisition, analysis, interpretation of the data, and drafting of the manuscript. LG-L analyzed histologically the removed appendixes. KX measured the Serum CRP. FV, BB, and FS participated in the study’s design. AK designed the study, was involved in interpretation of the data, the drafting of the manuscript, and revised it critically for the intellectual content until the final version was reached. All authors read and approved the final manuscript.

## References

[B1] Kozar RA Roslyn JJ Schwartz SI, Shires GT, Spencer FC The Appendix Principles of Surgery 1999 7 New York-London: The McGraw-Hill Companies Inc 1383 1393

[B2] Pal K Khan A Appendicitis: a continuing challenge J Pak Med Assoc 1998 48 7 189 192 10067019

[B3] Sartelli M Complicated intra-abdominal infections in Europe: preliminary data from the first three months of the CIAO Study World Journal of Emergency Surgery 2012 7 1 15 10.1186/1749-7922-7-15 22613202 PMC3444376

[B4] Khan MN Davie E Irshad K The role of white cell count and C-reactive protein in the diagnosis of acute appendicitis J Ayub Med Coll Abbottabad 2004 16 3 17 19 15631364

[B5] Groselj-Grenc M Repše S Vidmar D Derganc M Clinical and Laboratory Methods in Diagnosis of Acute Appendicitis in Children Croat Med J 2007 48 353 361 17589979 PMC2080535

[B6] Garcia Pena BM Cook EF Mandl KD Selective imaging strategies for the diagnosis of appendicitis in children Pediatrics 2004 113 24 28 Medline:14702442 10.1542/peds.113.1.24 14702442

[B7] Teepen HJ Zwinderman KA Comparison of CT and sonography in the diagnosis of acute appendicitis: a blinded prospective study AJR Am J Roentgenol 2003 181 1355 1359 14573433 10.2214/ajr.181.5.1811355

[B8] Lau WY Ho YC Chu KW Yeung C Leukocyte count and neutrophil percentage in appendicectomy for suspected appendicitis Aust N Z J Surg 1989 59 5 395 398 2730458 10.1111/j.1445-2197.1989.tb01593.x

[B9] Gurleyik E Gurleyik G Unalmişer S Accuracy of serum C-reactive protein measurements in diagnosis of acute appendicitis compared with surgeon's clinical impression Dis Colon Rectum 1995 38 12 1270 1274 10.1007/BF02049151 7497838

[B10] Mohammed AA Daghman NA Aboud SM Oshibi HO The diagnostic value of C-reactive protein, white blood cell count and neutrophil percentage in childhood appendicitis Saudi Med J 2004 25 9 1212 1215 15448768

[B11] Shakhatreh HS The accuracy of C-reactive protein in the diagnosis of acute appendicitis compared with that of clinical diagnosis Med Arh 2000 54 2 109 110 10934841

[B12] Kim-Choy N Shin-Wei L Clinical Analysis of the related factors in Acute Appendicitis Yale J Biol Med 2002 75 41 45 12074480 PMC2588704

[B13] Salem TA Molloy RG O’dwyer PJ Prospective study on the role of C-reactive protein (CRP) in patients with an acute abdomen Ann R Coll Surg Engl 2007 89 233 237 17394705 10.1308/003588407X168389 PMC1964747

[B14] Asfar S Safar H Khoursheed M Dashti H Al-bader A Would measurement of C-reactive protein reduce the rate of negative exploration for acute appendicitis? J R Coll Surg Edinb 2000 45 21 24 10815376

[B15] Kaiser S Mesas-Burgos C Soderman E Frenckner B Appendicitis in children – impact of US and CT on the negative appendectomy rate Eur J Pediatr Surg 2004 14 260 264 Medline:15343467 10.1055/s-2004-817841 15343467

[B16] Rosengren D Brown AF Chu K Radiological imaging to improve the emergency department diagnosis of acute appendicitis Emerg Med Australas 2004 16 410 416 Medline:15537403 10.1111/j.1742-6723.2004.00643.x 15537403

[B17] Jones K Pena AA Dunn EL Nadalo L Mangram AJ Are negative appendectomies still acceptable? Am J Surg 2004 188 748 754 Medline:15619494 10.1016/j.amjsurg.2004.08.044 15619494

[B18] Ponsky TA Huang ZJ Kittle K Eichelberger MR Gilbert JC Brody F Hospital- and patient-level characteristics and the risk of appendiceal rupture and negative appendectomy in children JAMA 2004 292 1977 1982 Medline:15507583 10.1001/jama.292.16.1977 15507583

[B19] Nwomeh BC Chisolm DJ Caniano DA Kelleher KJ Racial and socioeconomic disparity in perforated appendicitis among children: where is the problem? Pediatrics 2006 117 3 870 875 March 1 10.1542/peds.2005-1123 16510669

[B20] Albu E Miller BM Choi Y Lakhanpal S Murthy RN Gerst PH Diagnostic value of C-reactive protein in acute appendicitis Dis Colon Rectum 1994 37 49 51 10.1007/BF02047214 8287747

[B21] Davies AH Bernau F Salisbury A Souter RG C-reactive protein in right iliac fossa pain J R Coll Surg Edinb 1991 36 242 244 1941740

[B22] Grönroos JM Grönroos P A fertile-aged woman with right lower abdominal pain but unelevated leukocyte count and C-reactive protein: acute appendicitis is very unlikely Langenbecks Arch Surg 1999 384 437 440 10.1007/s004230050227 10552288

[B23] Andersson RE Hugander A Ravn H Offenbartl K Ghazi SH Nyström PO Repeated clinical and laboratory examinations in patients with an equivocal diagnosis of appendicitis World J Surg 2000 24 479 485 10.1007/s002689910076 10706923

[B24] Shoshtari MHS Askarpour S Alamshah M Elahi A Diagnostic value of Quantitative CRP measurement in patients with acute appendicitis Pak J Med Sci July - September 2006 22 3 300 303

[B25] Öztürk ZA Köklü S Erol MF Ylmaz FM Baar Ö Yüksel O Ylmaz G Ksack Yüksel B Serum adenosine deaminase levels in diagnosis of acute appendicitis Emerg Med J 2008 25 583 585 10.1136/emj.2007.054452 18723708

[B26] Pepys MB C-reactive protein fifty years on Lancet 1981 i 653 656 6110874 10.1016/s0140-6736(81)91565-8

[B27] Erkasap S Ates E Ustuner Z Sahin A Yilmaz S Yasar B Diagnostic value of interleukin-6 and Creactive protein in acute appendicitis Swiss Surg 2000 6 4 169 172 10.1024/1023-9332.6.4.169 10967943

[B28] Wu HP Lin CY Chang CF Chang YJ Huang CY Predictive value of C-reactive protein at different cutoff levels in acute appendicitis Am J Emerg Med 2005 23 4 449 453 10.1016/j.ajem.2004.10.013 16032609

[B29] Gronroos JM Gronroos P Leukocyte count and Creactive protein in the diagnosis of acute appendicitis Br J Surg 1999 86 4 501 504 10.1046/j.1365-2168.1999.01063.x 10215824

[B30] Eryilmaz R Sahin M Alimoglu O The value of C-reactive protein and leukocyte count in preventing negative appendectomies Ulus Treuma Derg 2001 713 142 145 11705213

[B31] Grönroos JMJU Do normal leukocyte count and C-reactive protein value exclude acute in children? Acta Paediatr 2001 90 6 649 5 11440098

[B32] Yokoyama S Takifuji K Hota T Matsuda K Nasu T Nakamori M Hirabayashi N Kinoshita H Yamaue H C-Reactive protein is an independent surgical indication marker for appendicitis: a retrospective study World J of Emergency Surgery 2009 4 36 10.1186/1749-7922-4-36 PMC2775727 19878592

[B33] Sinanan M Greenfield L Acute Abdomen and Appendix Surgery; Scientific Principles and Practice 2005 4 Philadelphia: J.B. Lippincot Company 1120 1142

[B34] Eriksson S Granstrom L Randomized controlled trial of appendicectomy versus antibiotic therapy for acute appendicitis Br J Surg 1995 82 166 169 10.1002/bjs.1800820207 7749676

[B35] Styrud J Eriksson S Nilsson I Ahlberg Haapaniemi S Neovius G Rex L Badume I Granstrom L Appendectomy versus antibiotic treatment in acute appendicitis; a prospective multicenter randomized trial World J Surg 2006 30 133 137 10.1007/s00268-005-0304-6 16736333

[B36] Exadactylos A Sadowski-Cron C Mader P Weissmann M Dinkel HP Negri M Zimmerman H Decision making in patients with acute abdominal pain at a university and at a rural hospital: does the value of abdominal sonography differ? World Journal of Emergency Surgery 2008 3 29 10.1186/1749-7922-3-29 18842129 PMC2572586

[B37] Leaper DJ Horrocks JC Staniland JR De Dombal FT Computer-assisted diagnosis of abdominal pain using “estimates” provided by clinicians Br Med J 1972 4 350 354 10.1136/bmj.4.5836.350 4629240 PMC1786557

[B38] Kessler N Cyteval C Gallix B Lesnik A Blayac PM Bruel JM Taourel P Appendicitis: Evaluation of sensitivity, specificity, and predictive values of US, Doppler US, and laboratory findings Radiology 2004 2 474 478 10.1148/radiol.2302021520 14688403

